# Early detection of acute kidney injury during the first week of the ICU

**DOI:** 10.1186/cc14365

**Published:** 2015-03-16

**Authors:** M Flechet, F Güiza, M Schetz, P Wouters, I Vanhorebeek, I Derese, J Gunst, G Van den Berghe, G Meyfroidt

**Affiliations:** 1 KU Leuven, Belgium

## Introduction

Acute kidney injury (AKI) is associated with increased morbidity and mortality in critically ill patients [1]. Early detection and treatment may improve outcome.

## Methods

A retrospective analysis of prospectively collected data from 2,158 patients without end-stage renal disease from the EPaNIC trial [2]. For early detection of AKI, defined according to the creatinine-based KDIGO guidelines [3], three multivariate logistic regression models (LR) were developed using data available at baseline (LR_B), upon ICU admission (LR_BA), and at the end of the first day in the ICU (LR_BAD1). In a subpopulation (*n *= 580) where plasma neutrophil gelatinaseassociated lipocalin (pNGAL), an early biomarker of AKI, was measured at ICU admission, the value of adding pNGAL to LR_BA and LR_BAD1 was assessed. The models were evaluated via bootstrapping, by comparing receiver operator characteristic (ROC) and decision curves.

## Results

Table 1 presents the performance of the models and admission pNGAL. Performance improved when predictions were made at a later time point, and was highest for LR_BAD1. Similar results were obtained in subgroups of septic and cardiac surgery patients. As an independent predictor, pNGAL alone did not perform better than a model using routine clinical data available upon admission. However, when combining pNGAL with LR_BA, predictive performance improved. The performance of LR_BAD1 was not improved by including pNGAL.

**Table 1:** Area under ROC curves for the different models.

LR_BA +			
pNGAL on		LR_BA on	pNGAL on
subpopulation LR_B with pNGAL (*n *= 2,158) (*n *= 528) (*n *= 528)		subpopulation LR_BA with pNGAL (*n *= 2,067) (*n *= 528)	subpopulationLR_BAD1 with pNGAL(*n *= 1,808)
0.64	0.73 0.70	0.76 0.76	0.82

## Conclusion

This study shows the potential of data-driven models based on routinely collected patient information for early detection of AKI during the first week of ICU stay. Although adding admission pNGAL to admission data improved early detection of AKI, this added value is lost upon inclusion of data from the first day of ICU.
